# Evolution of leadless pacemaker technology

**DOI:** 10.1093/eurheartjsupp/suae101

**Published:** 2025-03-24

**Authors:** Clemens Steinwender, Miguel A Leal

**Affiliations:** Department of Cardiology, Kepler University Hospital Linz, Medical Faculty, Johannes Kepler University, Altenbergerstrasse 69, 4040 Linz, Austria; Emory University School of Medicine, Emory Decatur Hospital, Electrophysiology Clinic, 2665 N Decatur Rd, Atlanta, GA 30322, USA

**Keywords:** Leadless cardiac pacing, Cardiac pacing, Pacemaker technology

## Abstract

Cardiac pacemakers have revolutionized the management of heart rhythm disorders since their introduction in the mid-20th century. They have played a crucial role in the management of several clinical conditions, from symptomatic bradyarrhythmia to electrical and/or mechanical dyssynchrony. Conventional cardiac pacemakers have significantly evolved over the last several decades. However, a critical limitation has been the presence of a generator pocket and endovascular leads connecting the pulse generator to cardiac tissue. While leads are essential to the pacemaker system by transmitting electrical impulses, they impose several significant risks that include venous thrombosis, device-associated endocarditis, and lead malfunction (mainly due to lead fracture, insulation failure, and macro- or micro-dislodgement), whereas the generator pocket can be the origin of a systemic device infection. The development of leadless pacemakers inaugurated a new era in cardiac pacing, eliminating many of the risks associated with traditional transvenous pacemakers. Leadless pacemakers are small, self-contained devices, which are fundamentally able to pace the heart without the need for a pocket and endovascular leads.

## Introduction

Cardiac pacemakers have truly revolutionized the management of heart rhythm disorders since their inception in the mid-20th century. These devices have played a crucial role in the management of several clinical conditions, from symptomatic bradyarrhythmias to electrical and/or mechanical dyssynchrony.

Conventional cardiac pacemakers have significantly evolved over the last several decades. However, a critical limitation has been the presence of a generator pocket and endovascular leads connecting the pulse generator to cardiac tissue.

While leads are essential to the pacemaker system by transmitting electrical impulses, they impose several significant risks that include venous thrombosis, device-associated endocarditis, and lead malfunction (mainly due to lead fracture, insulation failure, and macro- or micro-dislodgement), whereas the generator pocket can be the origin of a systemic device infection.^1,2^

The development of leadless pacemakers inaugurated a new era in cardiac pacing, eliminating many of the risks associated with traditional transvenous pacemakers. Leadless pacemakers are small, self-contained devices, which are fundamentally able to pace the heart without the need for endovascular hardware.^3–5^

### Historical background of cardiac pacemakers

Cardiac pacemakers were originally introduced in the 1950s, initially designed to treat patients with intermittent or permanent high-grade or complete heart block. The first pacemaker systems consisted of external devices that were connected to the heart via trans-thoracal wires.^6^ These devices were inherently large in volume and typically uncomfortable to the patient, but they quickly proved to be a life-saving therapy, by being able to electrically stimulate the heart with the goal of maintaining a perfusing rhythm.^7,8^

In 1958, the first fully implantable cardiac pacemaker was developed by two different groups in two continents: in Europe, the development and clinical implementation of the cardiac pacemaker are credited to Dr Åke Senning and Engineer Rune Elmqvist, and the surgery was performed in the Karolinska Institutet in Stockholm, Sweden. This pacemaker was encased in an epoxy resin and had an estimated battery life of 1–2 years.^9^

Across the Atlantic, Dr C. Walton Lilehei and Engineer Earl Bakken, in a similar demonstration of partnership between medical and engineering sciences, were responsible for the development and clinical application of an implantable cardiac pacemaker in the University of Minnesota, in Minneapolis, USA.^10^

While this was a revolutionary advancement in the management of cardiac rhythm disorders, pacemakers still fundamentally required endovascular leads, connecting the pulse generator to the heart. The following several decades were prolific in advancements in pacemaker technology, including longer battery life, smaller device size, expanded programmability options, and optimized lead design.

Despite these advancements, complications associated with the presence of a generator pocket and endovascular leads persisted, including infection, lead fracture, venous obstruction, and lead dislodgement.^1,2^ These issues prompted the exploration of alternative pacing systems that did not rely on leads, setting the stage for the development of leadless pacemakers.

### Conceptualizing leadless pacemakers

The idea of a leadless pacemaker was originally explored as early as the 1970s, but the technology was not feasible at the time due to limitations in battery miniaturization, device programming, and implantation techniques.^11^

The concept of a completely self-contained pacemaker that could be placed directly inside the heart without the need for external leads remained an elusive goal for decades. However, owing to significant advancements in microelectronics, battery technology, and materials science in the late 20th and early 21st centuries, the concept of a leadless pacemaker was brought much closer to reality.

Medical researchers and engineers, replicating a successful partnership that was originally responsible for the birth of cardiac pacing itself, began exploring the possibility of developing a small, self-contained device that could be safely implanted within the heart and also provide reliable long-term pacing support without the need for leads.

Those early efforts in leadless pacing were significantly limited by the challenges of creating a device that was small enough to be implanted in the heart via a catheter system, while still maintaining adequate battery life and performance over several years in order to make it a reasonable alternative to the already established traditional (transvenous) pacemaker systems. Additionally, methods for securely retrieving the device were not well developed at the time.

### The first leadless pacemaker: Nanostim

The first commercially available leadless pacemaker was called Nanostim. It was manufactured by St. Jude Medical, now Abbott, and it was introduced in the early 2010s.^12–14^

This device represented a significant leap forward in pacemaker technology. The Nanostim pacemaker was a cylindrical device approximately the size of a large vitamin pill, measuring ∼42 mm in length and weighing 2 g.

The Nanostim pacemaker was designed to be implanted directly into the right ventricle using a minimally invasive catheter-based procedure. The device contained all the necessary components essential for pacing, including a battery, a microprocessor, electrodes, and programmable pacing algorithms. It possessed an active fixation mechanism; i.e. it was secured to the right ventricular endocardium through a distal fixation helix that anchored the device in place.

The Nanostim pacemaker offered several advantages over traditional pacemakers:

- No leads: by eliminating leads, the risk of lead-related complications was significantly reduced (infection) or completely eliminated (lead fracture).
- Minimally invasive procedure: the device was implanted using a catheter percutaneously inserted through the femoral vein, allowing for short hospital stays and, in many cases, same-day discharge.
- Small size: the small size of the device eliminated the risk of complications related to the presence of a pacemaker pocket, such as infections or erosions.

Despite its potential to disrupt the field of cardiac pacing, the Nanostim faced several challenges, particularly regarding its implantation safety (with a relevant number of cases of cardiac perforation and tamponade reported among the original devices implanted) and its potential for retrieval.^13,14^

While the device was intentionally designed to be fully retrievable, the procedure proved to be technically challenging, with concerns regarding the long-term reliability of the retrieval mechanism, especially after several years of dwell time.^15,16^

Additionally, there were reports of premature battery depletion in some patients, which led to the voluntary recall of the device in 2016.^16^

The Nanostim experience was one of many cases that highlight the importance of careful pre-clinical and clinical data monitoring, as well as the need for further refinement of leadless pacemaker technology.

### The Micra transcatheter pacing system

Following the introduction of the Nanostim, Medtronic developed its own leadless pacemaker, known as the Micra transcatheter pacing system. The Micra, originally approved by the FDA in 2016, quickly gained recognition and widespread use, largely due to an established track record of safety, efficacy, and design improvements compared with its predecessor.^3,17^

**Figure 1:**
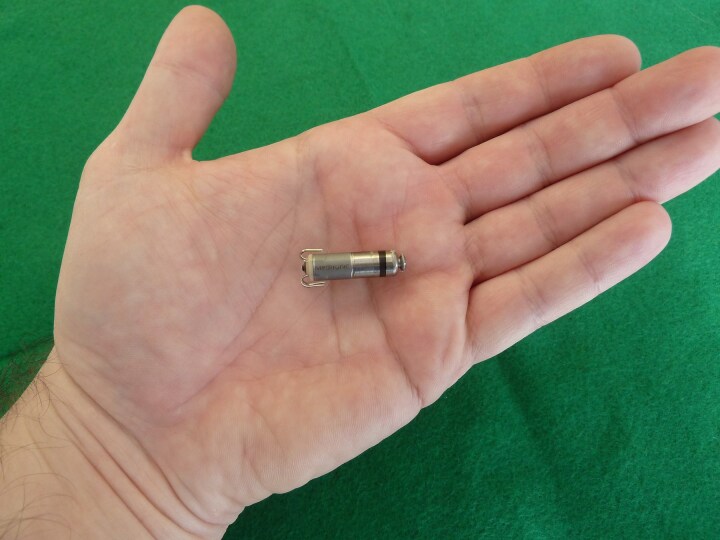
Micra VR leadless pacemaker, ^©^Clemens Steinwender, MD.

The Micra device measures ∼25.9 mm in length and weighs 2 g. Its shorter length allows for multiple placement options, including the basal and mid and apical interventricular septum (*Figure 1*). Notably, the first several implants had a preferential apical placement site, which proved to be inadequate given a sizable proportion of cardiac perforation and tamponade, with pre-clinical and early post-marketing series indicating a risk of 1–2%.^3,17,18^ Importantly, while traditional transvenous pacemaker leads do carry a similar risk of cardiac perforation, the morbidity and mortality rates associated with the latter are significantly lower, as less aggressive management (observation ± pericardiocentesis) is sufficient in the vast majority of cases.^19^ The Micra, however, required pericardiocentesis and/or surgical correction in a larger proportion of cases, likely due to the risks caused by the delivery catheter itself, as well as the device.^17,18^ In order to be adequately fixated to the right ventricular septal endocardium, a significant amount of forward force is indicated, oriented by biplanar contrast ventriculography, and if such force is applied while the catheter is not frankly septal in its orientation, free wall or inferior wall perforations may occur, resulting in the need for invasive corrective interventions. The enhanced delivery system (second generation) has a rounded catheter edge with more surface area to decrease tip pressure during device implant.^20^

The Micra had unique features, such as follows:

- Self-contained: like the Nanostim, the Micra is a fully self-contained device that does not require leads or a surgical pocket.
- Catheter-based implantation: the Micra is implanted using a minimally invasive catheter-based procedure through the femoral vein with a fixation mechanism using four nitinol tines.
- Reduced risk of infection: since the device is fully contained within the heart, the risk of infection is significantly reduced compared with transvenous pacemakers.^4,18,21,22^

The Micra device demonstrated excellent clinical outcomes in several large studies. In the Micra Investigational Device Exemption trial, the device showed a high procedure success rate, with 99.2% of patients experiencing successful implantation. The rate of major complications was significantly lower when compared with transvenous pacemakers (4% vs.7.4%).^3^ The safety profile of the Micra was particularly enhanced by virtually achieving the elimination of both lead- and pocket-related complications. Given the nature of the device, there was no risk of lead fracture, the dislodgement rate was significantly lower, and infections associated with the procedure and/or device were significantly reduced due to the absence of a subcutaneous pocket and the much smaller surface area of the device.^3,4,18^

Being worldwide the only commercially available leadless pacemaker over the years, most safety advancements in the implantation procedure as well as procedural risk assessment scores have been developed for the Micra device but can in the opinion of the authors be transferred to all other leadless pacemakers:

#### Personnel and facility requirements for leadless pacemaker implantation

While leadless pacemakers may have a lower risk of infection, the 2021 European Society of Cardiology guidelines strongly recommend implanting them under strict aseptic conditions in electrophysiology labs or operating rooms equipped with high-quality fluoroscopy.^23–25^ This ensures safe implantation and thorough assessment of the mechanical fixation. Recent data show that 78% of the 96 deaths associated with Micra implants were preceded by cardiac tamponade, often within the first hour, and surgically treated patients had a higher likelihood of survival.^26^ Therefore, each implanting centre should have a mandatory written protocol for managing tamponade. Pericardiocentesis or surgical intervention should be readily available, with on-site surgery being the preferred option. For transvenous pacemakers, a minimum annual volume of 25 or more leadless pacemaker implantations per centre is recommended, although definitive data are lacking. Operators should be trained in cardiac implantable electronic device implantation techniques and skilled in femoral vein access, large sheaths, and manoeuvres in the right atrium and ventricle.^25,27,28^

#### Risk score for pericardial effusion

Predictors of and a risk score (from 11 pre-procedural clinical parameters) for pericardial effusion in patients undergoing Micra leadless pacemaker implantations were recently determined by analysing a cohort of 2817 patients undergoing an implant attempt of a Micra device. The risk factors for pericardial effusion following Micra leadless pacemaker implantation are similar to those observed with traditional pacemaker procedures. These include advanced age, low body mass index, female gender, heart failure, prior heart attack, chronic obstructive pulmonary disease, lack of prior cardiac surgery, and haemodialysis. The risk of pericardial effusion can thus be reasonably predicted using routine clinical information. Additionally, multiple Micra deployments are linked to an elevated risk of pericardial effusion, especially in patients who already have an increased baseline risk.^29^

#### Implantation

**Figure 2:**
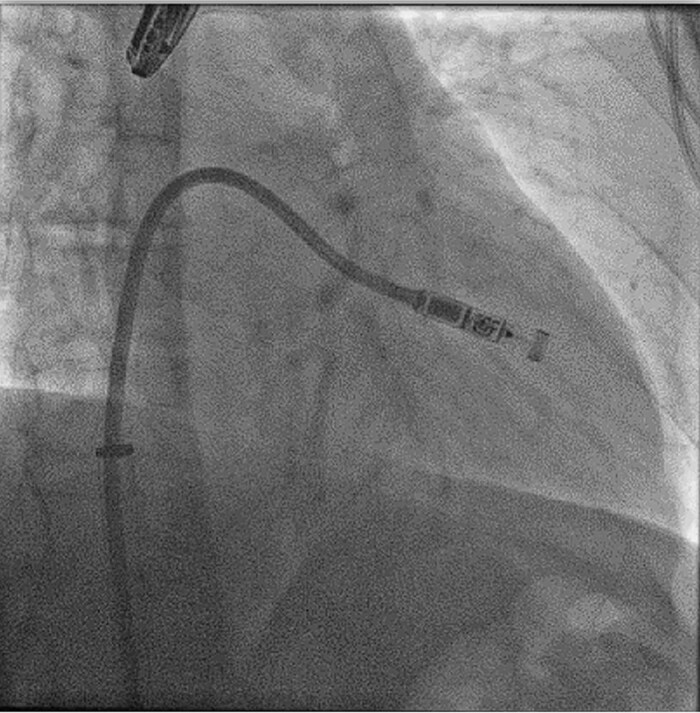
Femoral implantation of a Micra VR (right anterior oblique projection 35°), ^©^Miguel A. Leal, MD.

**Figure 3:**
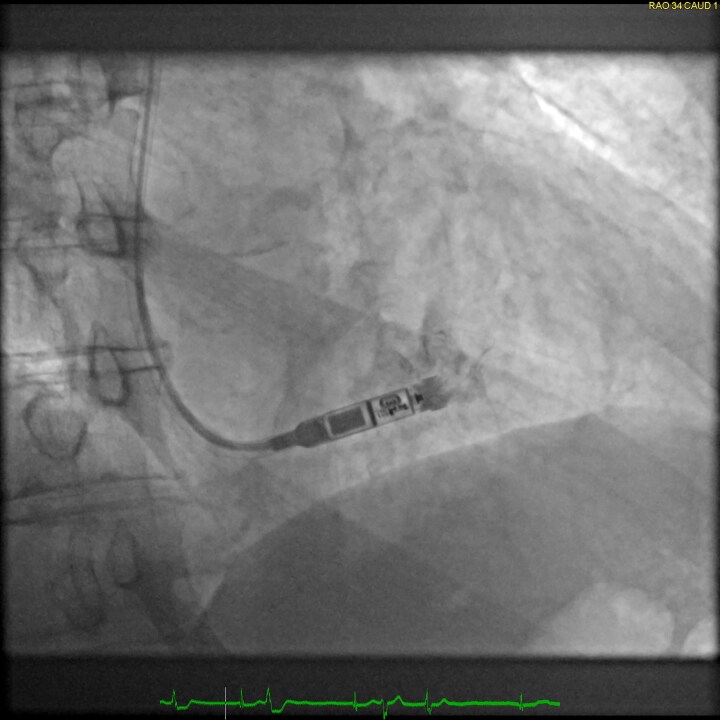
Jugular implantation of a Micra VR (right anterior oblique projection 34°), ^©^Clemens Steinwender, MD.

Leadless pacemakers are typically implanted through a right femoral venous approach using specialized delivery systems (*Figure 2*). Ultrasound-guided femoral vein puncture may reduce groin complications compared with the conventional approach, which can be beneficial for leadless pacemaker implantation and is meanwhile widely used.^30^ Alternative implantation techniques via the jugular vein have also been reported^31^ (*Figure 3*). In patients with left bundle branch block, a temporary backup pacing wire may be useful in case of a pre-existing right bundle branch block. The optimal location for device deployment is the mid-septum, as the free wall of the right ventricle should be avoided due to the risk of perforation.^3,4^ Determining the septal vs. free-wall position can be challenging using standard X-ray views, so individualized angulations or additional right ventriculography may facilitate proper positioning. Contrast injection through the delivery system should be considered to confirm wall contact.^25^

When implanting a subsequent leadless pacemaker without removing the previous one, cadaver studies suggest that up to three Micra devices can be accommodated simultaneously using traditional pacing sites, and human studies have demonstrated the safe implantation of two leadless pacemakers.^32^

#### Perioperative drug therapy

According to existing data for transvenous pacemakers, routine intravenous administration of antibiotics within 60–120 min of the implantation is routine, and is also recommended for leadless pacemakers. Additionally, as most of leadless pacemaker patients require oral anticoagulation for stroke prevention, the management of perioperative anticoagulation is an important consideration. Leadless pacemaker implantation is considered a minor bleeding risk procedure and can be performed with minimal interruption of direct oral anticoagulants, with a restart on the same day, 6 h after the procedure. For patients on vitamin K antagonists, the implantation can be safely performed when the International Normalized Ratio is within a low therapeutic range.^24,25^

#### Magnetic resonance imaging

Micra and Aveir (see below) leadless pacemakers are approved for full-body 1.5 T and 3 T magnetic resonance imaging (MRI). However, MRI scans should be avoided if the patient has elevated pacing thresholds. Prior to the MRI scan, both Micra and Aveir devices must be programmed to an MRI conditional mode. During the MRI, the patient's electrocardiogram and peripheral pulse should be continuously monitored by experienced personnel, with an external defibrillator/cardioverter with temporary pacing capabilities immediately available. After the MRI scan, the leadless pacemakers should be interrogated and reprogrammed as necessary.^25,33,34^

### The advent of the Aveir VR pacemaker

In 2022, Abbott was granted approval of its own leadless cardiac pacemaker, named the Aveir VR device. The pacemaker is longer than the Micra and possesses an active fixation helix, with enhanced safety features in comparison with its prototype (the Nanostim device). Its longer size allows for a relatively larger battery component, which has also led to estimated battery longevities that are projected to be numerically longer than the first generation of the Micra and similar to the second generation of Micra.^5,12–14^

**Figure 4:**
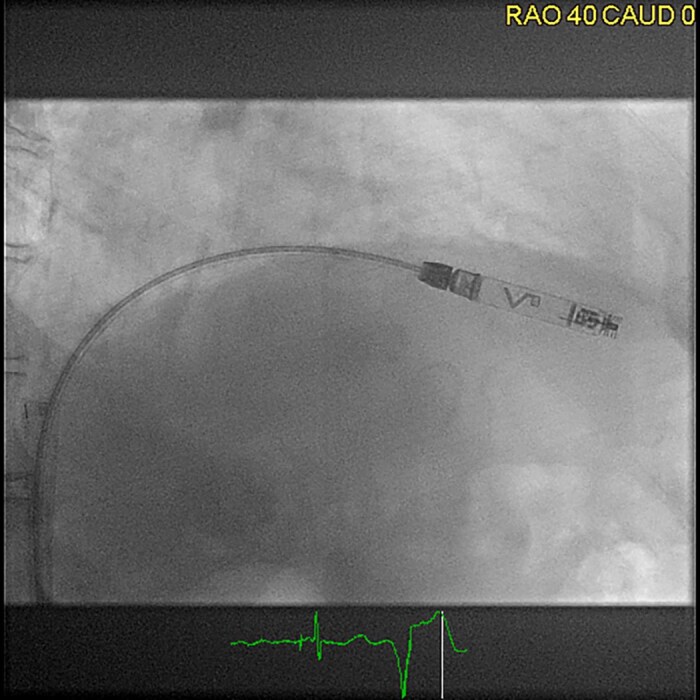
Femoral implantation of an Aveir VR (right anterior oblique projection 40°), ^©^Clemens Steinwender, MD.

The Aveir VR pacemaker is a small, cylindrical device, measuring around 38 mm in length and weighing ∼2.5 g. It is also implanted in the right ventricle using a catheter-based approach (*Figure 4*).

Key design features of the Aveir VR pacemaker system include the following:

- Retrievability: one advancement in the Aveir system is the inclusion of a dedicated retrieval catheter. This may prove to be advantageous in situations when the device’s battery is depleted, or if there is a need to upgrade or replace the pacemaker.^5,12–15^
- Extended battery life: the Aveir pacemaker incorporates a battery that is designed to last longer than first-generation leadless devices. This improvement addresses one of the concerns with leadless pacemakers, where battery replacement typically requires the implantation of a new device.
- Potential for dual-chamber pacing: Abbott has since been able to release the first-of-its-kind leadless pacemaker system (Aveir AR), which has brought to reality the concept of dual-chamber leadless cardiac pacing, which is discussed in more detail below.^6^

In a similar fashion to the Micra device, the Aveir leadless pacemaker is also implanted via a delivery catheter that is typically placed through either of the femoral vein. The procedure is generally performed under local anaesthesia combined with conscious (moderate) sedation, and most device implants take significantly less than an hour for the total procedural duration.^5,12–14^

The minimally invasive nature has led to a reduced recovery time and a lower risk of complications, again in a manner consistent with what has been noted with the Micra system over the last 7–8 years.^5,12–14^

Of note, owing to its longer profile, the Aveir VR pacemaker is designed for placement in the mid or apical segments of the interventricular septum, particularly given the theoretical risk of interaction with the tricuspid sub-valvular apparatus, if the device is implanted in the basal interventricular septal region.

### The next breakthroughs: synchronizing leadless ventricular pacing to the atrium and the case for leadless atrial pacing

While the first commercially available leadless pacemakers (Nanostim, Micra VR, and Aveir VR) undoubtedly represented a significant breakthrough in cardiac pacing technology, these devices were designed to be implanted directly into the right ventricle, therefore contributing to ventricular pacing.

The Micra AV device, which was released ∼4 years after the original Micra VR pacemaker system, contributed to a significant enhancement in leadless cardiac pacing by promoting P-synchronous ventricular pacing by the Micra AV pacemaker through an ingenious mechanism of detecting acceleration of blood flow into the ventricular chamber, and tracking the phenomenon associated with atrial contraction (the S4 phase of the cardiac cycle, which takes place in late diastole).

This enhanced algorithm, first described in the MARVEL clinical study, expanded the indication pool for leadless pacing to patients with an intact atrial (sinus) rhythm and some degree of atrioventricular block. It also allowed for a more ‘physiological’ delivery of cardiac pacing, potentially minimizing complications such as ‘pacemaker syndrome’, when ventricular pacing delivered asynchronously to the underlying atrial (typically sinus) rhythm may cause uncomfortable symptoms to the patient, such as lightheadedness, dyspnoea, head and neck fullness, and palpitations, among other clinical manifestation.^35,36^

**Figure 5:**
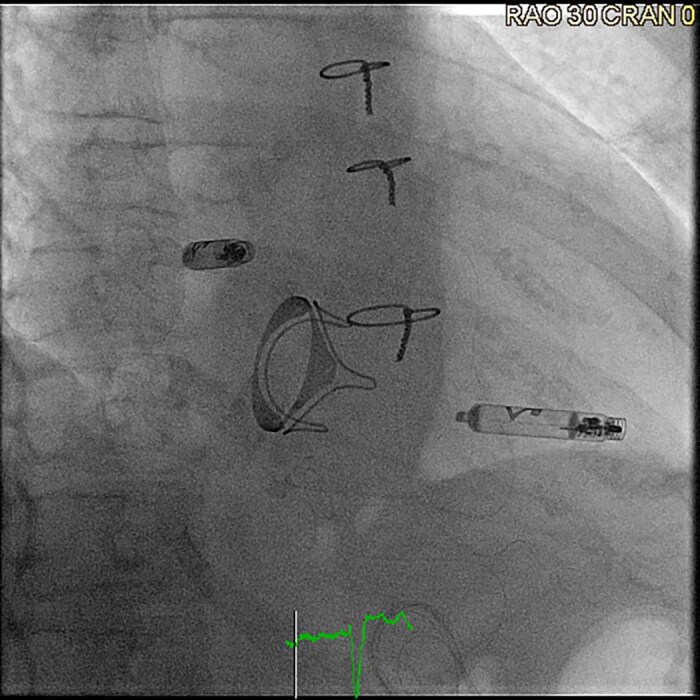
Aveir DR leadless pacemaker system after implantation (right anterior oblique projection 30°), ^©^Clemens Steinwender, MD.

However, pacing the atrial chamber, which is required for patients with sinus node dysfunction (‘sick sinus syndrome’), of cardioinhibitory syncope due to sinus arrest, for instance, remained a challenge. This was finally overcome in 2023, with the approval of the Aveir AR pacemaker system, developed by Abbott, being the second component of the revolutionary Aveir DR leadless pacemaker system^6^ (*Figure 5*).

This device has quickly gained adoption by many electrophysiologists given all the established benefits of leadless cardiac pacing in general. It is not identical to its ventricular counterpart, with a shorter length that allows for its placement in the right atrium, preferentially at the base of the right atrial appendage (or in alternative placement sites, such as the anterolateral surface of the right atrium), in a procedure guided by contrast injection of the right atrium and right atrial appendage, as well as monitoring of intra-procedural currents of injury and pacing impedance values, aimed to minimize the risk of cardiac perforation, particularly given the very thin characteristic of the right atrial myocardium.^6,37–39^

The median battery longevity of the currently commercially available leadless pacemakers, as specified by the manufacturers, is presented in *Table 1*.

**Table 1:** Estimated longevity of current commercially available leadless pacemakers

Device	Manufacturer	Estimated median longevity (years)
Micra VR1	Medtronic	12.3
Micra VR2	Medtronic	16.7
Micra AV1	Medtronic	10.8
Micra AV2	Medtronic	15.6
Aveir VR*	Abbott	17.6
Aveir AR*	Abbott	12.7

*Estimated longevity is based upon using devices individually; using the devices in dual-chamber mode estimates longevity at 6.4 years for Aveir AR and 11.3 years for Aveir VR.

### Technological advancements in leadless pacemakers

While the already approved miniaturized cardiac pacing devices certainly represent enormous milestones in the evolution of leadless pacing, ongoing research and development continue to drive further advancements in this field.

One of the primary challenges in developing leadless pacemakers has been balancing the need for a small, implantable device with the need for sufficient battery life and power efficiency, particularly as patients live longer lives, a dilemma similar to what has been faced by cardiac resynchronization therapy (CRT) devices over the last two decades.

Advancements in battery technology, microelectronics, and power management have made it possible to develop smaller, longer-lasting devices. New materials, such as lithium-polymer batteries, have helped improve battery life while maintaining a small device size. Additionally, advances in low-power integrated circuits and energy-efficient pacing algorithms have further extended the lifespan of leadless pacemakers.

Another important advancement in leadless pacemakers is the ability to wirelessly communicate with external programming devices. Older pacemaker systems required transcutaneous communication with external programmers, but modern devices, including some leadless pacemakers, use wireless radiofrequency communication, which allows physicians to monitor and adjust the pacemaker settings in a more time-efficient manner.

An improvement that is expected to occur in the near future for the Aveir system and that is already available for the Micra system is wireless communication that enables remote monitoring, where the pacemaker can transmit data to a home monitoring system, allowing for continuous patient surveillance by the device clinic where the patient is enrolled. Remote monitoring has a track record of more than 20 years with proven evidence of improved patient outcomes by promoting early diagnosis and intervention of conditions such as atrial tachyarrhythmias, other forms of significant tachyarrhythmias (including ventricular tachycardia) and possible device and/or lead malfunction.^40–42^

One of the challenges with leadless pacemakers has been the concern about the difficulty of retrieving and/or replacing the devices. While traditional pacemakers can be easily replaced when the battery depletes, leadless pacemakers are more difficult to retrieve due to their intrinsic form of implantation within the heart.^15,43^

Retrieval techniques using specialized catheters designed to capture and remove the device have been designed, but they depend on a significant amount of device exposure available to the operator at the time of the procedure, likely inversely proportional to the device’s dwell time.^15,43–45^ In cases where complete or near-complete device ‘endothelialization’ has occurred, which may require imaging modalities such as intracardiac echocardiography for confirmation, rather than removing the old device, a new pacemaker should be implanted alongside the existing one when the original system’s battery depletes. This ‘stacking’ of devices has been shown to be safe and effective in most patients, with the understanding that most right ventricular chambers can accommodate two or even three leadless cardiac pacemakers.^32^

### Future directions in leadless pacing

The field of leadless pacemakers is still in its early stages, and there is considerable potential for future advancements. Several areas of ongoing research and development are likely to shape the future of leadless pacing.

Currently, leadless pacemakers are limited to right atrial or right ventricular pacing. This leaves the issue of CRT or conduction system pacing (CSP) unresolved in terms of applying leadless pacing technology to patients who may benefit from either of those pacing modalities.

Left ventricular pacing has been evaluated with the WISE-CRT system (made by EBR Systems, Sunnyvale, CA, USA), which has been studied both in Europe and in the USA. This unique system utilizes an endocardial left ventricular pacing unit or electrode, which is also delivered via catheter either in anterograde (via transseptal puncture) or retrograde (via a transaortic catheterization) approach. This system includes a pulse generator and a transmitter that are implanted first in order to locate the appropriate implanting site for the left ventricular electrode.^46,47^

Another application that will benefit from integration with leadless pacing technologies is the modular system developed by Boston Scientific, which combines the Empower leadless pacemaker with the Emblem subcutaneous defibrillator (S-ICD), allowing for anti-tachycardia pacing therapies to be delivered by the leadless pacemaker in an effort to minimize the need for cardioversion or defibrillation shocks delivered by the S-ICD system. This system is not yet approved by the FDA, but the published data regarding its clinical feasibility and safety profile suggest that this technology will become clinically available in the very near future.^48^

The placement of leadless cardiac pacemakers in specific areas associated with CSP, particularly the His bundle region (accessed via the right atrium, potentially from a superior approach) or the left bundle branch area region (which will require a different delivery method and fixation mechanism in comparison with the currently available devices), is an object of active research efforts at the present time, including delivering dual-chamber pacing using a single device, and poised to become the next frontier in this rapidly evolving field.^49^

Finally, another exciting area of research involves integrating leadless pacemakers with other emerging technologies, such as biological pacing. Biological pacing involves the use of gene therapy or stem cells to restore normal heart rhythm without the need for an electronic device. While still in early experimental stages, biological pacing has the potential to complement or even replace traditional pacemakers in the future.^50^

As leadless pacemakers continue to evolve, it is critically important to continue to assess their long-term performance and biocompatibility. Ensuring that these devices remain safe and effective over many years will be critical for their widespread adoption, and the possible replacement of most (if not all) forms of lead-based devices, thus revolutionizing the short- and long-term safety profile of cardiac pacing therapy. A more frequent use of leadless pacemakers may possibly result in lower device costs.

## Conclusion

The evolution of leadless cardiac pacemakers represents a remarkable achievement in the field of cardiology. From the early days of large, external devices to the development of self-contained leadless systems like Micra and Aveir, pacemaker technology has made significant strides in both prolonging and improving the quality of the lives of many patients with bradyarrhythmias.

While leadless pacemakers have already shown great promise in reducing complications associated with traditional transvenous pacemakers, there is still significant room for advancement. Ongoing research and development in device miniaturization, battery power efficiency, multi-site pacing, and biological pacing will continue to shape the future of this technology.

As the field of leadless pacemakers continues to evolve, it holds the potential to further improve patient outcomes, reduce complications, and transform the way we manage heart rhythm disorders in the future.

## Data Availability

No new data were generated or analysed in support of this research.
